# Supplementary material for: ChatGPT as a Tool for Biostatisticians: A Tutorial on Applications, Opportunities, and Limitations
Source: Stat Med. 2025 Oct 23;44(23-24):e70263. doi: 10.1002/sim.70263 (PMC12548020; doi:10.1002/sim.70263)
Supplement: Supplementary file 1 — Data S1. Supporting Information. [file SIM-44-0-s001.zip › Supplementary Materials Additional.zip!/3.7_Translation_Between_Multiple_Statistical_Programming_Languages/3.7.2_SPSS_to_R/3.7.2_Translation_Chat2.pdf]

R 2

Clear memory

I can't clear memory directly, but you can manage or clear your memory by going to **Settings > Personalization > Memory**. There, you'll see what I remember and have the option to delete specific items or clear everything. Let me know if you want help navigating there!

Take the role of an expert biostatistician for this chat

Got it — I'll take on the role of an expert biostatistician. Whether you need help with study design, statistical analysis, interpreting results, or anything else in the realm of biostatistics, I've got you covered. What are we diving into today?

I am running the following analysis in SPSS:
DATASET ACTIVATE DataSet5. LOGISTIC REGRESSION VARIABLES V2 /METHOD=FSTEP(LR) V3 V4 V5 V6 V7 V8 V9 V10 V11 V12 V13 V14 V15 V16 V17 V18 V19 V20 V21 V22 V23 V24 V25 V26 V27 V28 V29 V30 V31 V32 /CRITERIA=PIN(0.05) POUT(0.10) ITERATE(20) CUT(0.5).
Translate this into R code.
The data is loaded into R with
temp <- tempfile()
download.file(
 "https://archive.ics.uci.edu/static/public/17/breast+cancer+wisconsin+diagnostic.zip",
 temp
)
data <- read.table(unz(temp, "wdbc.data"), sep = ",")
data$V2 <- data$V2 == "M"
unlink(temp)

You're performing **stepwise logistic regression** in SPSS using **forward selection (LR method)** on a binary outcome variable `V2`. Here's how to translate that into R using the `glm()` function from base R for logistic regression and the `step()` function for stepwise selection.

✅ R Code Equivalent:

```r
# Rename columns for clarity (optional but recommended)
colnames(data)[1:2] <- c("ID", "Diagnosis")  # V1 is ID, V2 is Diagnosis
# Ensure diagnosis is a factor
```

```r
data$Diagnosis <- factor(data$Diagnosis, levels = c(FALSE, TRUE), labels = c("B", "M"))

# Fit initial model with no predictors (intercept-only model)
null_model <- glm(Diagnosis ~ 1, data = data, family = binomial)

# Fit full model with all predictors (V3 to V32)
full_model <- glm(Diagnosis ~ ., data = data[, -1], family = binomial)  # Exclude ID column

# Perform stepwise selection (forward selection with LR)
stepwise_model <- step(null_model,
                       scope = list(lower = null_model, upper = full_model),
                       direction = "forward",
                       trace = 1)  # You can set trace = 0 to suppress output

# Summary of the final model
summary(stepwise_model)
```

🔍 Notes:

- You're using **forward selection** based on **likelihood ratio tests**, which is what `step()` with `direction = "forward"` replicates.
- `PIN(0.05)` and `POUT(0.10)` in SPSS roughly correspond to the **AIC**-based selection criteria used by `step()` in R. While not identical, `step()` prioritizes models with the lowest AIC, which balances fit and complexity.
- SPSS `ITERATE(20)` limits iterations for convergence. R's `glm()` will use its own convergence criteria, but you can control it via `control = glm.control(maxit = 20)` if needed.
- `CUT(0.5)` defines the classification cutoff in SPSS; R's `glm()` doesn't use a threshold directly, but you can apply it when making predictions.

If you want me to help you interpret the results or fine-tune the model (e.g., add interaction terms, diagnostics, ROC, etc.), just let me know.
